# Musculoskeletal Pain and Hip Muscle Length Among Undergraduates: A Longitudinal Study of 1 Year

**DOI:** 10.3390/medicina62081548

**Published:** 2026-08-12

**Authors:** Janan Abbas, Noa Reif, Kamal Hamoud

**Affiliations:** Department of Physical Therapy, Zefat Academic College, Zefat 13206, Israel; noar@medical360.org (N.R.); kamalh@zefat.ac.il (K.H.)

**Keywords:** musculoskeletal pain, hip muscle length, students, history of pain

## Abstract

*Background and Objectives*: Musculoskeletal pain (MSP) is highly prevalent among undergraduate students and may originate during early adulthood, increasing the risk of chronicity later in life. Modifiable factors such as reduced muscle flexibility and prolonged sedentary behavior have been proposed as contributors; however, longitudinal evidence among student populations remains scarce. This study aimed to prospectively examine changes in MSP prevalence and hip muscle length over one year and to identify independent predictors of MSP. *Materials and Methods*: A prospective longitudinal study was conducted among 62 first-year undergraduate students assessed at baseline and at one-year follow-up. MSP was evaluated using a modified Standardized Nordic Questionnaire, and demographic data including physical activity and sitting behavior were recorded. Hamstring and iliopsoas muscle length were measured by passive straight-leg raise and modified Thomas tests, respectively. Changes over time were analyzed using paired *t*-tests and McNemar tests. Multivariable logistic regression was also performed to identify predictors of MSP at follow-up. *Results*: Most participants were female (79%, *n* = 49), with a mean age of 25.8 ± 6.0 years and body mass index of 23.6 ± 4.4 kg/m^2^. Significant reductions in hamstring (*p* < 0.001; d = −0.55 to −0.70) and iliopsoas muscle length (*p* < 0.001; d = −1.32 to −1.46) were observed over one year. Health science students manifested a significant decrease in hamstring length over time compared to their counterparts in other programs (*p* ≤ 0.002). MSP prevalence increased across all body regions, particularly in the low back (46.8% to 71.0%) and cervical spine (46.8% to 61.3%). Baseline MSP was the only independent predictor of MSP at follow-up (upper quadrant: OR = 12.89, 95% CI: 2.38–69.65, *p* = 0.003; lower quadrant: OR = 6.35, 95% CI: 1.39–28.90, *p* = 0.018). *Conclusions:* A significant increase in the prevalence of MSP was observed alongside a reduction in hip muscle length among undergraduate students over a one-year period. The findings suggest that students enrolled in health science programs are more susceptible to musculoskeletal alterations, whereas baseline MSP is a strong predictor of future symptoms.

## 1. Introduction

Musculoskeletal pain (MSP) is a leading contributor to disability and reduced quality of life worldwide and represents one of the most common reasons for healthcare utilization [1,2]. MSP encompasses pain arising from muscles, joints, tendons, ligaments, and spinal structures, with low back pain, neck pain, and lower-limb pain among the most frequently reported conditions [3,4]. Notably, low back pain is the leading cause of years lived with disability globally, underscoring the substantial public health burden associated with MSP [2,5]. Although MSP is often associated with older adults and occupational populations, accumulating evidence indicates a high prevalence among young adults, particularly undergraduate students [6,7,8,9].

University students are exposed to multiple physical, behavioral, and psychosocial stressors during a critical stage of life. Previous studies have demonstrated associations between MSP and prolonged sitting, extensive screen use, poor ergonomics, insufficient physical activity, sleep disturbances, and elevated academic stress [10,11,12,13,14,15]. Contemporary pain models further suggest that MSP is influenced by a complex interaction of biological, psychological, and social factors rather than by biomechanical mechanisms alone [14]. Accordingly, MSP is highly prevalent among university students, with reported prevalence rates ranging from approximately 40% to 80%, depending on the population studied, body region assessed, and recall period [10,11,12,13,16,17,18,19,20]. Likewise, it has been reported that musculoskeletal pain experienced during early adulthood tracks into later life and is associated with an increased risk of chronic pain and long-term functional limitations [21].

In recent years, increasing attention has been directed toward the role of muscle flexibility, particularly of the hip musculature in the development and maintenance of MSP [22,23,24]. Hip muscles play a central role in lumbopelvic stability and lower-limb biomechanics [25,26]. Reduced muscle extensibility, especially of the iliopsoas and hamstrings, has been associated with altered pelvic alignment, increased lumbar spine loading, and compensatory movement patterns [26,27,28]. These alterations may increase susceptibility to musculoskeletal disorders such as low back pain, hip dysfunction, and lower-extremity overuse injuries [29,30,31,32]. Prolonged sitting, a common behavior among university students, combined with high levels of stress, has been linked to adaptive shortening of hip flexors and reduced flexibility of the posterior chain, potentially contributing to postural deviations and movement dysfunction [20,26,33,34].

Despite growing interest in this area, most existing studies are cross-sectional and have primarily focused on athletic or clinical populations, limiting both generalizability and the ability to infer temporal relationships [35,36]. Longitudinal evidence examining changes in hip muscle length and their relationship with MSP in undergraduate populations remains scarce. We believe that cumulative behavioral exposures over an academic year may be sufficient to produce measurable musculoskeletal adaptations. Additionally, the longitudinal study design allowed us to evaluate temporal changes beyond the short-term alterations commonly reported in cross-sectional studies, which may have important implications for early identification and preventive strategies.

The aims of this study were to (1) examine longitudinal changes in musculoskeletal pain prevalence and hip muscle length over one academic year, (2) identify factors associated with MSP at follow-up, and (3) determine whether academic program was associated with changes in hip muscle length or MSP prevalence.

Based on previous evidence, we hypothesized that both MSP prevalence and hip muscle length would change over the one-year follow-up period. Given the multifactorial nature of MSP, including the potential influence of lifestyle, psychosocial, and environmental factors, this study was designed primarily to explore these longitudinal changes and their potential associations.

## 2. Materials and Methods

### 2.1. Study Design and Participants

This longitudinal study was conducted among first-year undergraduate students enrolled in full-time bachelor’s degree programs at Zefat Academic College, Israel. The study included a one-year follow-up period.

**Figure 1:**
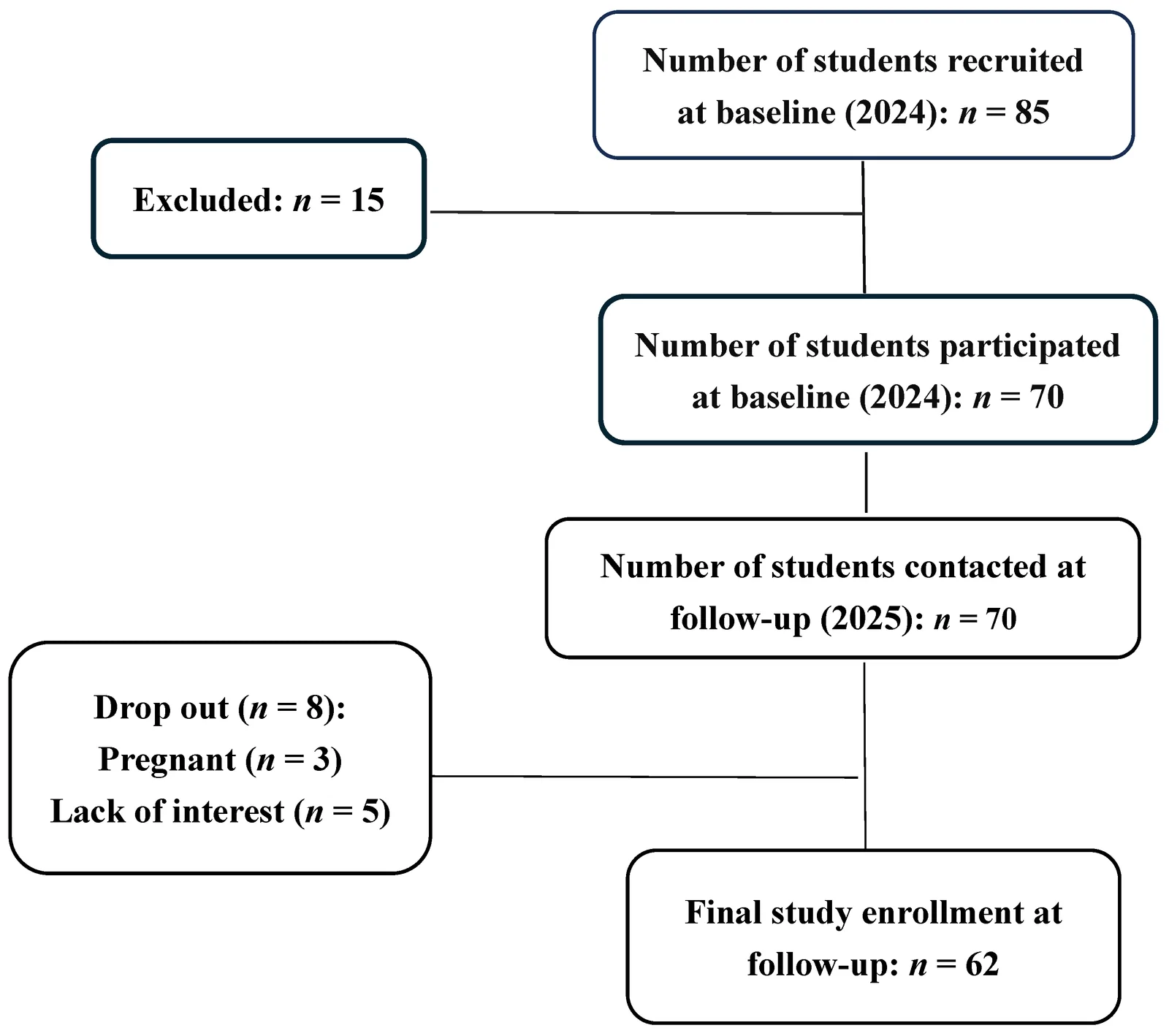
Flow chart of the study sample within baseline and follow-up.

Participants were recruited between May and June 2024 through institutional communication channels (e.g., the college website and social media platforms). Eligible participants were students aged ≥18 years who were enrolled in one of five academic programs (Nursing, Physiotherapy, Occupational Therapy, Social Work, and Art and Literature). Recruitment across multiple programs was intended to enhance sample heterogeneity. The exclusion criteria were (1) pregnancy; (2) history of spinal or lower extremity surgery; (3) diagnosed neuromusculoskeletal disorders; and (4) structural deformities of the spine or thoracic cage. A total of 70 students were initially recruited, of whom 62 completed the one-year follow-up assessment (Figure 1).

All participants provided written informed consent prior to participation. The study was conducted in accordance with the Declaration of Helsinki and was approved by the Institutional Research Ethics Committee of Zefat Academic College (approval number: 2-2024).

### 2.2. Data Collection and Outcome Measures

Data collection and physical assessments were performed at two points: baseline (May–June 2024) and follow-up (May–June 2025).

Musculoskeletal pain (MSP) was assessed using a hard copy of the modified version of the validated Standardized Nordic Questionnaire [37]. Participants were asked whether they had experienced pain, aching or discomfort in any of these specific body regions (e.g., cervical, thoracic, lumbar spine, upper and lower extremities) during the preceding three months and the past week. Participants who reported pain in both the preceding 3 months and the past week, regardless of its severity, were considered positive for MSP. Pain intensity was measured following the visual analog scale (VAS) [38].

MSP was also categorized into two regions: (1) upper quadrant, which includes the cervical and thoracic spine and upper extremities, and (2) lower quadrant, which involves the lumbar spine and lower extremities. Changes in MSP status over the follow-up period were classified into three categories: 1 = improved, 2 = no change, and 3 = worsened. Sociodemographic and lifestyle-related variables were also collected and included age, sex, smoking status, physical activity, and sedentary behavior [39,40]. Physical activity was recorded as a dichotomous variable (yes/no), based on self-reported engagement in aerobic exercise. Sedentary behavior was assessed by reporting the daily sitting time and categorized as ≤5 h or >5 h per day. Stress-related variables (e.g., academic and personal stress) were assessed using a four-point Likert scale (very high, high, low, none) referring to the preceding month [31]. Anthropometric measurements were obtained using a calibrated digital scale (Shekel H150-5, Tziporit industrial area, Isreal). Body mass index (BMI) was calculated as weight (kg) divided by height squared (m^2^).

Hamstring and iliopsoas muscle lengths were assessed following the study of Abbas et al. [24]. Hamstring length was measured using the passive straight-leg raise test (PSLR), with the participant in a supine position and the contralateral limb stabilized. The hip was passively flexed while maintaining knee extension until a firm end-feel and a tolerable stretch sensation were reached. Iliopsoas muscles were evaluated using the modified Thomas test (MTT), in which participants assumed a supine position while maintaining maximal flexion of the contralateral hip and knee, allowing the tested limb to hang freely over the edge of the examination table [24]. The reported minimal detectable changes (MDC95) for the PSLR and MTT are approximately 6.6–10° and 4.17°, respectively [41,42,43].

All measurements were performed by a single trained examiner (RN) who was blinded to participants’ MSP status to reduce measurement bias. Each measurement was repeated three times per limb, and the mean value was used for analysis.

### 2.3. Statistical Analysis

A post hoc power analysis was conducted based on the final sample size (*n* = 62), accounting for 8 dropouts (11%), with a two-sided α level of 0.05 and statistical power set at 0.80. The study was adequately powered to detect moderate-to-large effect sizes (r > 0.40). Additionally, baseline characteristics (e.g., age and hip muscle length) were comparable between participants who completed the study and those lost to follow-up, suggesting a low risk of attrition bias. The intraclass correlation coefficient (ICC) was calculated to assess the intra-tester and inter-tester reliability of muscle flexibility measurements over 5–7 day intervals, based on repeated assessments in a subsample of 15 participants. The normality of continuous variables (e.g., age, weight, and muscle length) was evaluated using the Kolmogorov–Smirnov test. Descriptive statistics are used to summarize participant characteristics: continuous variables are presented as mean ± standard deviation (SD), while categorical variables are reported as frequencies and percentages.

Changes in hip muscle length between baseline and follow-up were analyzed using paired-samples *t*-tests. Between-group differences (health science vs. non-health science students) in muscle length changes were assessed using independent-samples *t*-tests. Changes in the prevalence of musculoskeletal pain (MSP) over time were evaluated using the McNemar test. Given the sample size (*n* = 62) and the number of outcome events (48–49 MSP cases at follow-up in both the upper and lower body quadrants), the number of predictors included in the multivariable logistic regression models was carefully restricted to avoid overfitting and unstable estimates. Although the number of events was relatively high, the overall sample size remained modest, which may compromise model stability if too many predictors are included. In accordance with methodological recommendations regarding the events-per-variable (EPV) ratio (commonly ≥10 events per predictor), the present data support the inclusion of approximately 4–5 predictors per model. Therefore, to maintain an adequate EPV ratio and enhance model robustness, only a limited set of clinically and theoretically relevant variables was selected a priori (e.g., baseline MSP, muscle length measurements). This parsimonious approach reduces the risk of overfitting, limits inflation of effect estimates, and improves the generalizability and interpretability of the findings. Spearman’s rank correlation analysis was also used to examine the associations between changes in MSP status and enrollment in a health sciences program, as well as changes in muscle length. Statistical significance was set at *p*-values < 0.05.

## 3. Results

The intra-tester and inter-tester reliability results (ICCs) for measuring the hamstring and iliopsoas lengths were very high: 0.995 to 0.985 and 0.992 to 0.942, respectively. As we used a relatively small sample size (starting with 70 and ending with 62 students), we can reliably detect only moderate-to-large effects (≥0.4).

### 3.1. Demographic Characteristics

The majority of participants were female (79%, *n* = 49), and 71% (*n* = 44) were enrolled in health science programs (Table 1). The mean age of the participants was 25.8 ± 6.0 years, and the mean body mass index (BMI) was 23.6 ± 4.4 kg/m^2^. Approximately 12.9% (*n* = 8) of participants were smokers, and 51.6% (*n* = 32) reported engaging in aerobic physical activity. Prolonged sitting (>5 h/day) was reported by 51.6% (*n* = 32) of the sample. High to very high levels of study-related stress were reported by 83.9% (*n* = 52) of the participants.

### 3.2. Changes in Hip Muscle Length

Significant reductions in both hamstring and iliopsoas muscle lengths were observed over the one-year follow-up period (*p* < 0.001) (Table 2). The effect sizes (Cohen’s *d*) indicated moderate-to-large reductions in hamstring length (*d* = −0.55 to −0.70) and large reductions in iliopsoas length (*d* = −1.32 to −1.46). These findings suggest that the decline in muscle length was moderate for the hamstrings and substantial for the iliopsoas over time. The observed changes in hamstring and iliopsoas muscle length exceeded the reported minimal detectable change (MDC_95_) values for both the passive straight leg raise (PSLR: 6.6–10°) and the modified Thomas test (MTT: 4.17°), suggesting that the changes were greater than the measurement error and are likely to represent true changes over the one-year follow-up. In addition, the large reduction in hip muscle length over time was significantly greater among health science students compared to other students only for hamstring length (*p* ≤ 0.002; *d* = −0.78 to −1.01) (Table 3). Notably, baseline characteristics were comparable between health science students and students from other academic programs, except for age. Health science students were significantly younger than students from other programs (23.5 ± 2.6 vs. 28.2 ± 9.9 years, *p* = 0.005).

### 3.3. Musculoskeletal Pain (MSP)

The prevalence of musculoskeletal pain increased across all assessed body regions over time (Table 4). This trend was also noted in pain intensity (VAS). The largest increases were observed in low back pain (from 46.8% to 71.0%) and cervical spine pain (from 46.8% to 61.3%). Substantial increases were also noted in the thoracic region (24.2% to 46.8%) and in the wrist and hand (21.0% to 43.5%). Smaller increases were observed in lower-limb regions, including the hip, knee, and ankle.

Analysis by body quadrant demonstrated a statistically significant increase in MSP prevalence over time in both the upper quadrant (64.5% to 77.4%, *p* = 0.021) and lower quadrant (54.8% to 79.0%, *p* < 0.001) (Table 5). Spearman’s rank correlation analysis demonstrated a mild positive association between enrollment in a health science program and worsening MSP status over the one-year follow-up (r = 0.264, *p* = 0.038). However, no significant associations were observed between changes in muscle length and changes in MSP status.

Multivariable logistic regression analysis identified baseline MSP as the only significant predictor of MSP at follow-up. Participants reporting MSP at baseline had substantially higher odds of experiencing MSP at follow-up in both the upper quadrant (OR = 12.89, 95% CI: 2.38–69.65, *p* = 0.003) and lower quadrant (OR = 6.35, 95% CI: 1.39–28.90, *p* = 0.018) (Table 6). Neither baseline muscle length nor self-reported study-related stress was independently associated with MSP at follow-up.

## 4. Discussion

To the best of our knowledge, this is among the first studies to longitudinally examine changes in hip muscle length and musculoskeletal pain among undergraduate students. The longitudinal design provides insight into changes in these musculoskeletal characteristics over a one-year period, although direct comparisons with previous studies are limited. Given the predominance of female participants and the relatively small sample size, caution should be applied when generalizing these findings to populations with different sex distributions.

The results demonstrated an increase in and a high prevalence of musculoskeletal pain (MSP) alongside a reduction in hip muscle length among first-year undergraduate students over a one-year follow-up period at Zefat Academic College.

The findings also revealed a significant reduction in the length of both the iliopsoas and hamstring muscles bilaterally, with moderate-to-large effect sizes (Cohen’s d ranging from −0.55 to −1.46). The reduction was more pronounced in the iliopsoas muscles (d = −1.32 to −1.46), suggesting greater susceptibility of this muscle to change over time. Importantly, the magnitude of the observed changes in hamstring and iliopsoas muscle length exceeded the reported MDC_95_ values for the PSLR (6.6–10°) and MTT (4.17°). Therefore, the changes observed over the one-year follow-up are unlikely to be attributable to measurement error alone and likely reflect valid changes in muscle length. Previous studies have suggested that specific manual therapy interventions such as structural integration may induce short-term postural changes, improve hip mobility, and reduce compensatory movement patterns [44,45]. Therefore, it is plausible that sustained postural habits and behavioral factors, including prolonged sitting and stress-related movement behavior, over the course of one year, may contribute to adaptive changes in hip muscle length. This could be supported by the concept of musculoskeletal tissues being responsive to changes in posture and psychosocial behavior over 1 year of follow-up [31,46,47]. Posture alterations could be developed due to the high prevalence of prolonged sitting reported by participants, with approximately 52% indicating sitting durations exceeding five hours per day. Previous studies have demonstrated associations between prolonged sitting and alterations in muscle length and increased stiffness [34,48,49,50]. Several physiological mechanisms may explain the indirect relationship between prolonged sitting and reduced muscle extensibility. Sustained static postures and reduced movement may increase passive muscle stiffness through changes in connective tissue properties, muscle perfusion, and metabolic activity, resulting in greater passive resistance and reduced flexibility [51,52,53,54,55,56,57]. However, these mechanisms remain speculative in the context of the present study, as no direct physiological measures were obtained.

Our findings also demonstrated an increase in and a high prevalence of MSP across all assessed body regions over time. This outcome agrees with others [16,17,18]. The most notable increases were observed in the lower back (46.8% to 71%) and cervical region (46.8% to 61.3%), followed by the wrist and hand and the thoracic spine. These findings are consistent with previous studies indicating that the lower back and neck are the most affected regions in young adult populations [11,13,15,19]. Analysis of MSP by body quadrant (upper vs. lower) revealed a statistically significant increase over time in both regions. Notably, Spearman’s rank correlation analysis revealed a mild positive correlation between changes in MSP status over time and enrollment in a health science program. However, no significant correlation was observed between changes in MSP status and changes in muscle length. We also observed a more pronounced reduction in hamstring muscle length among students enrolled in health science programs than among their peers from other academic programs. These findings are consistent with previous studies reporting a higher prevalence of MSP among health science students than their counterparts [58,59,60]. This increased prevalence has been attributed to the demanding nature of health science curricula, which often involve prolonged screen time, sedentary behavior, clinical training, and elevated levels of academic stress, all of which have been associated with the development of low back pain [12,61,62]. Although sitting duration and perceived stress were comparable between academic programs in the present study, our findings reinforce the concept of MSP as a multifactorial condition. Therefore, factors beyond those assessed in this study may also contribute to MSP [13,15,19].

The observed increase in MSP may be partly explained by prolonged sitting and sustained postures, which are associated with increased mechanical loading, postural strain, and muscle fatigue. Prolonged sitting has also been linked to broader musculoskeletal consequences, including neck and shoulder pain [63,64]. Gupta et al. (2015) reported a positive association between sitting time and low back pain intensity [65]. It has further been suggested that university students may be at increased risk of musculoskeletal disorders due to prolonged screen time, sustained sitting behavior, and high levels of academic stress [66].

The decreased hip flexor muscle length observed in the present study may contribute to anterior pelvic tilt, thereby affecting lumbar spine alignment and increasing spinal loading and pain [67,68]. These adaptations, together with other factors such as psychological stress, may partly explain the concurrent increase in MSP prevalence and reduction in muscle length observed over the one-year follow-up. However, no significant association was found between changes in muscle length and changes in MSP status. This finding suggests that changes in muscle length and the development of MSP may be influenced by different biological, behavioral, and psychosocial pathways operating at the individual level. In addition, the onset and progression of MSP may occur gradually over time and may not correspond directly to changes in muscle length. Finally, these variables represent different constructs, as muscle length was assessed objectively, whereas MSP was based on self-reported symptoms.

The multivariable logistic regression analysis demonstrated that the presence of MSP at baseline (in both the upper and lower body quadrants) was the only significant predictor of MSP at the one-year follow-up. This finding is consistent with the existing literature indicating that a history of musculoskeletal pain is a strong predictor of future symptoms [69,70]. For instance, a recent longitudinal study among health science students reported that a prior history of pain was significantly associated with the occurrence of low back pain over a two-year follow-up period [69]. However, the magnitude of the observed associations should be interpreted cautiously due to the instability of the estimates, as reflected by the wide confidence intervals. In addition, the Nagelkerke R^2^ values for the regression models ranged from 0.47 to 0.55, indicating that approximately 50% of the variance in MSP at follow-up was explained by the variables included in the models. This suggests that additional unmeasured factors such as ergonomic exposures, sleep disturbances, and other lifestyle or psychosocial variables may also contribute to the development and persistence of MSP. The outcome that muscle length at baseline was not associated with musculoskeletal pain (MSP) at follow-up (logistic regression analysis) does not rule out a potential relationship between these variables. Instead, it suggests that muscle length alone may not be a major determinant of MSP in this population, and that other biological, behavioral, or psychosocial factors may have a greater influence. Furthermore, this finding should be interpreted with caution because the number of variables included in the logistic regression model was deliberately limited to reduce the risk of overfitting, which may have restricted the ability to identify additional independent predictors.

Limitations of the study. This study should be interpreted considering several limitations. First, the relatively small sample size limited statistical power and subgroup analyses, restricted the number of predictors included in the regression models, and may have contributed to overfitting and imprecise estimates, as reflected by the wide confidence intervals. Second, the sample was derived from a single academic institution, consisted predominantly of female participants, and had a relatively higher mean age than undergraduate cohorts reported in other countries (24 years vs. approximately 19 years at program entry), which may limit the generalizability of the findings. Additionally, reliance on self-reported questionnaire data introduces the potential for bias, including recall bias and the influence of unmeasured socioeconomic factors. Third, although musculoskeletal pain (MSP) is a multifactorial condition, several potentially relevant lifestyle-related variables, such as sleep quality, sitting posture, psychosocial stress, and duration of mobile device use, were not assessed, limiting a more comprehensive understanding of contributing factors. Fourth, the one-year follow-up period may have allowed the influence of unmeasured changes in participants’ lifestyle, academic workload, physical activity, or health status, which could have affected muscle length and MSP outcomes. Although this duration was selected to capture potential long-term musculoskeletal adaptations over an academic year, residual confounding cannot be excluded. Finally, pain assessment was relatively limited, as detailed characteristics of MSP (e.g., frequency, duration, severity, disability, and pain type) were not systematically evaluated, thereby restricting insights into the heterogeneity and clinical impact of symptoms.

### Clinical Implications

The findings of this study highlight the importance of early identification and monitoring of musculoskeletal impairments among undergraduate students. The observed reduction in hip muscle length, alongside the increased prevalence of musculoskeletal pain (MSP), particularly among health science students, suggests that multiple variables such as postural and behavioral factors may be associated with musculoskeletal health in this population. In addition, screening individuals with a history of MSP may be valuable, as baseline pain was identified as a strong predictor of future symptoms. Future large-scale, multicenter studies using adequately powered longitudinal and interventional designs are needed to determine whether interventions targeting modifiable behavioral and postural factors can prevent or reduce musculoskeletal pain (MSP) among undergraduate students.

## 5. Conclusions

In this longitudinal study of a predominantly female sample, a significant increase in the prevalence of musculoskeletal pain (MSP) was observed alongside a reduction in hip muscle length among undergraduate students over a one-year period. The findings suggest that students enrolled in health science programs are more susceptible to musculoskeletal alterations, whereas baseline MSP is a strong predictor of future symptoms. Future large-scale, multicenter studies are needed to identify the modifiable factors associated with MSP in this population.

## Figures and Tables

**Table 1:** Participant characteristics at 1-year follow up (*n* = 62).

Variable	*n* (%)/or Mean ± SD
Male	13 (21)
Female	49 (79)
Mean age (year)	25.8 ± 6
Mean BMI (kg/m^2^)	23.6 ± 4.4
Smoking	8 (12.9)
Physical activity (yes)	32 (51.6)
Prolonged daily sitting:	
up to 5 h	30 (48.4)
>5 h	32 (51.6)
* Study-related stress:	
Very high–high	52 (83.9)
Little–none	10 (16.1)
Health science	44 (71)
Other	18 (29)

BMI—Body mass index, SD—standard deviation. * Stress-related variable appeared alone, as this variable was the most common compared to other types of stress.

**Table 2:** Comparison of hip muscle length between baseline and one-year follow-up of the study sample (paired-samples *t*-test).

Muscle	Mean ± SD Baseline (*n* = 62)	Mean ± SD Follow-Up (*n* = 62)	T (61)	*p* Value	Cohen’s *d*
Hamstrings rt	92.3 ± 18	81 ± 20	−5.48	<0.001	−0.70
Hamstrings lt	91.3 ± 17	81 ± 19	−4.31	<0.001	−0.55
Iliopsoas rt	11.3 ± 7	−0.8 ± 8	−11.47	<0.001	−1.46
Iliopsoas lt	12.4 ± 7	0.98 ± 8	−10.37	<0.001	−1.32

SD—standard deviation, rt—right, lt—left. Negative T values and Cohen’s d indicate a reduction in muscle length over time.

**Table 3:** Comparison of changes in hip muscle length between health science and other students (independent-samples *t*-test).

Muscle	Muscle Length Decreases (Mean ± SD)		T (df)	*p* Value	Cohen’s *d*
	Health Science (*n* = 44)	Others (*n* = 18)			
Hamstrings rt	−13.88 ± 15	−2.63 ± 10	−3.23 (45.58)	**0.002**	−0.78
Hamstrings lt	−14.10 ± 17	1.83 ± 10	−4.43 (52)	**<0.001**	−1.01
Iliopsoas rt	−11.89 ± 8	−10.25 ± 6	−0.83 (40.78)	0.413	−0.208
Iliopsoas lt	−12.17 ± 8	−9.55 ± 8	−1.10 (32.83)	0.279	−0.303

SD—standard deviation, rt—right, lt—left. Negative T values indicate a reduction in muscle length. Cohen’s d represents effect size.

**Table 4:** Prevalence (%) and intensity (mean **±** SD) of musculoskeletal pain across body regions at the baseline and follow-up.

Body Region	MSP at Baseline % (*n*)	VAS at Baseline ± SD	MSP at the Follow-Up % (*n*)	VAS at Follow-Up ± SD
Cervical	46.8 (29)	3.2 ± 1.8	61.3 (38)	5.3 ± 2
Thoracic	24.2 (15)	2.9 ± 1.6	46.8 (29)	5.3 ± 1.9
Shoulder	35.5 (22)	3 ± 1.6	45.2 (28)	5.8 ± 2.4
Elbow	1.6 (1)	4 ± 0	14.5 (9)	4.6 ± 1.8
Wrist and fingers	21 (13)	3.6 ± 1.9	43.5 (27)	4.5 ± 1.8
LBP	46.8 (29)	3.7 ± 2	71 (44)	5.2 ± 2.3
Hip	8.1 (5)	4.2 ± 3.3	16.1 (10)	5.2 ± 2.1
knee	22.6 (14)	3.8 ± 1.8	29 (18)	5.1 ± 2.8
Ankle and foot	11.3 (7)	3.6 ± 2.6	16.1 (10)	5 ± 2.1

SD—standard deviation, VAS—visual analogue scale.

**Table 5:** Changes in musculoskeletal pain prevalence by body quadrant (upper vs. lower) over time (McNemar test).

Quadrant	MSP Baseline % (*n*)	MSP Follow-Up % (*n*)	*X*^2^ (1)	*p* Value
Upper	64.5 (40)	77.4 (48)	5.33	0.021
Lower	54.8 (34)	79 (49)	11.27	<0.001

**Table 6:** Multivariable logistic regression analysis of factors associated with musculoskeletal pain at one-year follow-up by each quadrant separately.

**Upper Quadrant Pain**			
Nagelkerke R^2^ = 0.465			
**Variable**	**Adjusted OR**	**CI 95%**	***p*** ** Value**
Upper quadrant pain (baseline)	12.89	2.38–69.65	**0.003**
Mean hamstrings (baseline)	1.06	0.99–1.13	0.079
Mean iliopsoas (baseline)	0.95	0.85–1.06	0.333
BMI (baseline)	0.86	0.74–1.06	0.180
Study-related stress	0.41	0.08–2.22	0.303
**Lower Quadrant Pain**			
Nagelkerke R^2 ^ = 0.552			
Lower quadrant pain (baseline)	6.35	1.39–28.90	**0.018**
Mean hamstrings (baseline)	1.03	0.99–1.07	0.187
Mean iliopsoas (baseline)	1.05	0.99–1.12	0.137
BMI (baseline)	0.96	0.87–1.06	0.369
Study-related stress	1.51	0.50–4.52	0.463

BMI—Body mass index, CI—confidence intervals.

## Data Availability

Datasets are available to download on request. Requests should be directed to the corresponding author.
